# Clinical Outcomes of Patients Treated for *Candida auris* Infections in a Multisite Health System, Illinois, USA

**DOI:** 10.3201/eid2605.191588

**Published:** 2020-05

**Authors:** Kellie Arensman, Jessica L. Miller, Anthony Chiang, Nathan Mai, Joseph Levato, Erik LaChance, Morgan Anderson, Maya Beganovic, Jennifer Dela Pena

**Affiliations:** Advocate Lutheran General Hospital, Park Ridge, Illinois, USA (K. Arensman, M. Beganovic, J. Dela Pena);; Advocate South Suburban Hospital, Hazel Crest, Illinois, USA (J.L. Miller);; Advocate Trinity Hospital, Chicago, Illinois, USA (J.L. Miller);; Swedish Covenant Hospital, Chicago (A. Chiang);; Advocate Christ Medical Center, Oak Lawn, Illinois, USA (N. Mai, J. Levato);; Advocate Illinois Masonic Medical Center, Chicago (E. LaChance);; Advocate Condell Medical Center, Libertyville, Illinois, USA (M. Anderson);; Advocate Good Shepherd Hospital, Barrington, Illinois, USA (M. Anderson)

**Keywords:** Candida auris, candidiasis, candidemia, antimicrobial resistance, susceptibility, CLABSI, BSI, nosocomial infections, bacteria, bacterial infections, Illinois, United States

## Abstract

*Candida auris* is an emerging fungal pathogen that is typically resistant to fluconazole and is known to cause healthcare-associated outbreaks. We retrospectively reviewed 28 patients who had >1 positive culture for *C. auris* within a multisite health system in Illinois, USA, during May 2018–April 2019. Twelve of these patients were treated as inpatients for *C. auris* infections; 10 (83%) met criteria for clinical success, defined as absence of all-cause mortality, *C. auris* recurrence, and infection-related readmission at 30 days from the first positive culture. The other 2 patients (17%) died within 30 days. Most patients (92%) were empirically treated with micafungin. Four (14%) of 28 total isolates were resistant to fluconazole, 1 (3.6%) was resistant to amphotericin B, and 1 (3.6%) was resistant to echinocandins. Our findings describe low rates of antifungal resistance and favorable clinical outcomes for most *C. auris* patients.

*Candida auris* is an emerging, multidrug-resistant, healthcare-associated fungal pathogen that was first reported in Japan in 2009 and has now been isolated on 6 continents (*1*–*9*). *C. auris* has been identified as the causative pathogen in various invasive fungal infections, including bloodstream infections (*2*,*4*), and is associated with outbreaks across healthcare settings (*6*,*10*). Risk factors for *C. auris* infection are similar to other *Candida* infections including prolonged hospitalization, abdominal surgery, diabetes mellitus, intensive care unit (ICU) admission, use of central venous and urinary catheters, immunocompromising conditions, chronic kidney disease, and exposure to broad-spectrum antibiotic and antifungal agents (*10*–*13*). Investigations in the Chicago, Illinois, USA, area have found a high prevalence of *C. auris* colonization at ventilator-capable skilled nursing facilities (*14*) and have shown higher rates of *C. auris* colonization among patients who are mechanically ventilated, have a gastrostomy tube, or have a urinary catheter (*15*). Reported mortality rates attributable to invasive *C. auris* infection range from 30% to 59% globally (*13*,*16*) and from 22% to 57% in the United States (*8*,*10*,*17*).

*C. auris* isolates are often resistant to fluconazole and have variable susceptibility to other antifungal agents (*13*,*16*). The Centers for Disease Control and Prevention (CDC) currently recommends echinocandins as empiric therapy for suspected or confirmed *C. auris* infections (*18*). However, recent reports have indicated reduced susceptibilities to echinocandins and suggest that resistance might be inducible under antifungal pressure (*8*,*16*).

Previous reports of *C. auris* infections and outbreaks have largely focused on epidemiologic information, and data on treatment strategies and clinical outcomes are limited (*6*,*8*,*10*,*16*–*21*). We report microbiologic data for *C. auris* isolates from a multisite health system in Illinois and an assessment of clinical outcomes for patients treated for *C. auris* infections.

## Methods

This study is a retrospective cohort analysis of patients at 8 hospitals within a single health system located in the Chicago metropolitan area. We included all patients >18 years old who had >1 positive culture for *C. auris* from any anatomic site during January 1, 2008–April 30, 2019; we excluded pregnant patients, prisoners, and patients <18 years of age. If a patient had multiple positive cultures for *C. auris*, we included only the first positive culture per hospital encounter. Patients who died before culture result were not included in clinical success evaluation. The study received a non–human subjects research determination from the Advocate Aurora Health Institutional Review Board.

The microbiology laboratory for this system primarily uses matrix-assisted laser desorption/ionization time-of-flight (MALDI-TOF) mass spectrometry (Vitek MS, bioMérieux, https://www.biomerieux.com) for organism identification. Because our database does not include *C. auris*, for isolates not identified by our MALDI-TOF mass spectrometry system, we used Vitek 2 version 8.01 (bioMérieux), which we have been using since December 2017. We sent isolates identified as *C. haemulonii, C. duobushaemulonii*, and *Candida* spp. not identified by Vitek 2 to the Illinois Department of Public Health for additional testing using MALDI-TOF mass spectrometry or genomic sequencing to rule out misidentification of *C. auris*. During June 2018–April 2019, we sent all isolates identified as *C. auris* to the Illinois Department of Public Health, which forwarded them to CDC for whole-genome sequencing. We performed antifungal susceptibility testing with colorimetric microdilution by using Sensititer YeastOne YO9 (TREK Diagnostic Systems, https://www.trekds.com). Because no *C. auris* susceptibility breakpoints have been established, we used tentative breakpoints published by CDC for interpretation in this study (*22*).

We performed manual chart review for all patients. We evaluated patient charts for demographic information, infection source, culture source and susceptibilities, empiric and definitive therapy, length of hospital and ICU stay, clinical success, and reports of adverse events associated with treatment for *C. auris* infection. We defined clinical success as the absence of 30-day all-cause mortality, 30-day recurrence of the same organism, and 30-day infection-related readmission. We identified adverse drug events associated with antifungal therapy by reviewing patient laboratory results and progress notes from healthcare providers.

## Results

We evaluated records from 8 hospitals from the period of January 1, 2008, through April 30, 2019, for *C. auris* isolates. Cultures were obtained as part of routine clinical practice. A total of 28 patients from 5 hospitals had >1 positive culture for *C. auris* during the study period. We included 28 *C. auris* isolates in this study (the first isolate from our health system was collected in May 2018). Members of the cohort had a median age of 70 years (interquartile range 62*–*78 years), and most (20 [71%]) patients were men. Most (26 [93%]) patients were admitted from a skilled nursing facility; 1 patient was transferred from another hospital, and 1 was admitted from the community. Nine (75%) patients required chronic mechanical ventilation, and 6 (21%) were receiving hemodialysis through a central line. Most isolates were cultured from blood (12 [43%]) or urine (10 [36%]). The median time from admission to collection of the first culture positive for *C. auris* was 0.14 days (interquartile range 0*–*0.88 days). The average hospital stay for inpatients was 12 days. Thirteen patients (46%) were admitted to an ICU; the average ICU stay was 3 days.

MICs for the 28 *C. auris* isolates (Table 1) showed that 4 (14%) were resistant to fluconazole, 1 (3.6%) was resistant to amphotericin B, and 1 (3.6%) was resistant to echinocandins, according to tentative *C. auris* breakpoints published by CDC (*22*). One isolate was resistant to fluconazole, amphotericin B, and echinocandins. This isolate was from a patient who was considered to be colonized with *C. auris* in the urine and did not receive antifungal therapy.

**Table 1:** MICs of 28 *Candida auris* isolates from patients treated for *C. auris* infections in a multisite health system, Illinois, USA*

Antifungal drug	MIC, µg/mL													
	0.015	0.03	0.06	0.12	0.25	0.5	1	2	4	8	64	128	256	>256
Anidulafungin			3.6	64.3	28.6					3.6				
Caspofungin		3.6	25.0	42.9	25.0			3.6						
Micafungin			50.0	39.3	3.6	3.6				3.6				
Fluconazole								39.3	35.7	10.7	3.6	3.6	3.6	3.6
Itraconazole		7.1	32.1	39.3	17.9	3.6								
Posaconazole	10.7	50.0	25.0	14.3										
Voriconazole	42.9	21.4	21.4			3.6			10.7					
Amphotericin B			10.7				82.1	3.6						
Flucytosine			17.9	71.4	10.7									

*Values are percentage of isolates having the MIC shown. Shaded values are considered resistant on the basis of Centers for Disease Control and Prevention tentative *C. auris* breakpoints (*22*).

Twelve patients (43%) were treated as inpatients for *C. auris* infections (Table 2). Of those patients who were not treated with antifungal therapy, 2 were evaluated in the emergency department and were discharged back to their skilled nursing facility in stable condition before their blood cultures results were available, 3 patients died before culture results were available, and 11 were considered to be colonized with *C. auris*. Of those patients who were treated for *C. auris* infections, most were found to have a central line–associated bloodstream infection (CLABSI) (7 [59%]), whereas others were treated for catheter-associated urinary tract infection (2 [17%]), skin and skin structure infection (2 [17%]), and other bloodstream infection (BSI) (1 [8%]). All patients who were treated for *C. auris* infections were under the care of a physician specialized in infectious diseases.

**Table 2:** Demographic and clinical characteristics of patients treated for *Candida auris* infections in a multisite health system, Illinois, USA*

Patient age, y/sex	Culture source (infection type)	Empiric treatment	Definitive treatment	Treatment duration	Outcome	Comments
83/M	Urine (CA-UTI)	Micafungin 100 mg IV every 24 h	Micafungin 100 mg IV every 24 h	5 d	Clinical success	Trach to vent patient with dementia. Urine culture earlier in admission showed 10,000–50,000 CFU *C. auris*, but thought to be colonization and was not treated. Repeat urine culture showed >100,000 CFU *C. auris*, and patient was treated.
56/M	Blood (CLABSI)	Micafungin 100 mg IV every 24 h	Fluconazole 200 mg per PEG every 24 h	15 d	Clinical success	Trach to vent patient with ESRD on HD with tunneled catheter, also had a PICC. Both lines were removed.
73/M	Blood (CLABSI)	Micafungin 100 mg IV every 24 h	Micafungin 100 mg IV every 24 h	17 d	Clinical success	Trach to vent patient with ESRD on HD with tunneled catheter, chronic osteomyelitis of the coccyx. *C. auris* from culture of HD line at SNF. Tunneled catheter removed.
64/F	Blood (CLABSI)	Micafungin 100 mg IV every 24 h	Micafungin 100 mg IV every 24 h	26 d	Died	Trach to vent patient with ESRD on HD with chest port and PICC for TPN. Lines removed. 42 d of therapy planned; patient readmitted for presumed septic shock and died on day 26 after being switched to comfort care. No growth of any organisms in cultures on readmission.
61/M	Catheter tip	Micafungin 100 mg IV every 24 h	Micafungin 100 mg IV every 24 h	21 d	Clinical success	Trach patient with ESRD on HD with tunneled catheter admitted for fungemia. Started on micafungin before admission. Line removed. Azole not used because of concomitant amiodarone.
74/M	Urine (CA-UTI)	Micafungin 100 mg IV every 24 h	Micafungin 100 mg IV every 24 h	Unknown	Clinical success	Trach to vent patient. Patient transferred to SNF before culture finalized; duration of micafungin to be determined by SNF.
74/F	Blood (CLABSI)	Micafungin 100 mg IV every 24 h	Fluconazole 400 mg PO every 24 h	21 d	Clinical success	SNF patient on chronic TPN for enterocutaneous fistulas, history of line infections and infective endocarditis. Persistently fungemic for 4 d until tunneled central line was removed.
50/F	Abdominal wound	Micafungin 100 mg IV every 24 h	Micafungin 100 mg IV every 24 h	10 d	Clinical success	Patient with obesity, diabetes, and chronic abdominal/groin ulcers hospitalized for DKA; receives wound care at home. Ulcers underwent debridement; *C. auris*, CoNS, and *Corynebacterium* grew from operative cultures.
78/M	Blood	Fluconazole 400 mg IV every 24 h	Itraconazole 200 mg per PEG every 24 h	14 d	Clinical success	Trach to vent after cardiac arrest, midline POA for hypotension and hypoxia. Midline thought to be source. Discharged to hospice, but continued antifungal therapy. Lost to follow-up.
79/M	Blood (CLABSI)	Micafungin 100 mg IV every 24 h	Micafungin 100 mg IV every 24 h	5 d	Died	Trach, ESRD on HD with tunneled catheter. Blood culture also showed growth of *Proteus mirabilis.* Died from septic shock after switching to comfort care. Repeat blood cultures showed no growth.
78/F	Hip synovial fluid	Micafungin 100 mg IV every 24 h	Micafungin 100 mg IV every 24 h	6 d	Clinical success	ESRD on HD with tunneled catheter, DM, prosthetic mitral valve, treated for drainage from hip after hip replacement 3 mo prior, had onset of septic shock after I&D procedure. *C auris* isolated from hip aspirate. Antifungal treatment stopped after 6 d because *C. auris* was a suspected contaminant. Died in hospital >30 d after *C auris* isolation.
82/M	Blood (CLABSI)	Micafungin 100 mg IV every 24 h	Micafungin 100 mg IV every 24 h	14 d	Clinical success	Patient with functional quadriplegia after CVA. Trach, PEG, PICC, and chronic foley catheter POA. PICC removed.

*CA-UTI, catheter-associated urinary tract infection; CFU, colony forming units; CLABSI, catheter-associated urinary tract infection; CoNS, coagulase negative *Staphylococci*; CVA, cerebral vascular accident; DKA, diabetic ketoacidosis; DM, diabetes mellitus; ESRD, end-stage renal disease; HD, hemodialysis; I&D, incision and debridement; PEG, percutaneous endoscopic gastrostomy; PICC, peripherally inserted central catheter; POA, present on admission; SNF, skilled nursing facility; TPN, total parenteral nutrition; trach, tracheotomy; vent, ventilator.

Patients were empirically treated with micafungin (11 [92%]) or fluconazole (1 [8%]). Of those patients empirically treated with micafungin, most were being treated for CLABSI (7 [64%]), followed by catheter-associated urinary tract infection (2 [18%]) and skin and skin structure infection (2 [18%]). For definitive treatment, patients received micafungin (9 [75%]), fluconazole (2 [17%]), or itraconazole (1 [8%]). Of those patients who received an azole as definitive treatment, all were being treated for BSIs. Treatment duration ranged from 5 to 26 days (mean 14 days). The only adverse event noted was an increase in aspartate aminotransferase from 25 to 91 U/L in 1 patient being treated with fluconazole. Fluconazole was continued, and the patient was discharged on fluconazole to complete their treatment course. 

Ten (83%) patients met criteria for clinical success. No patients were found to have *C. auris* recurrence or infection-related readmission within 30 days of first positive culture. Two (17%) patients died within 30 days of first positive culture; both were being treated for CLABSIs.

## Discussion

We report patient characteristics and microbiologic data for 28 patients with >1 positive culture for *C. auris*. We also describe the clinical outcomes for 12 patients treated for *C. auris* infections. Our observed mortality rate of 17% is lower than previously reported worldwide. A meta-analysis of 742 patients from 16 countries found an all-cause mortality rate of 30% (*13*). Our lower mortality rate might be a result of empirically selecting echinocandin therapy, to which >95% of our isolates were susceptible. Empiric selection of fluconazole for treatment of *C. auris* BSI was recently described in a case series in India (*19*). *C. auris* is often resistant to fluconazole, and lack of effective empiric therapy can result in poor outcomes. Because echinocandins are commonly empirically selected for treatment of candidemia in the United States, our lower mortality rates might be attributable to lower MICs in our geographic region. For instance, investigators of an outbreak in New York, New York, found a 45% mortality rate among 51 patients with BSIs at 90 days (*10*). The mortality rate in our study was 25% among patients with BSI. A separate case series from Brooklyn, New York, reported a 22% in-hospital mortality rate among a cohort of 9 patients with BSIs, which is similar to our findings (*17*). Small sample size and variations in underlying conditions might confound mortality rate comparisons, although another possible explanation for the lower mortality rate in our study is differences in antifungal resistance.

Previous reports of *C. auris* collections have noted substantially higher rates of antifungal resistance than what was observed in our cohort. In a grouping of 54 isolates from 5 countries, 93% were resistant to fluconazole, 35% to amphotericin B, and 7% to echinocandins (*16*). The first isolates from the United States demonstrated a similar pattern; 86% were resistant to fluconazole, 43% to amphotericin B, and 3% to echinocandins (*18*). An even higher rate of fluconazole resistance of 98% was noted in New York, New York (*10*). Drug-resistance mechanisms are genetically encoded, and some resistance mutations are linked to specific geographic clades (*16*). An epidemiologic investigation of 133 *C. auris* isolates from the United States showed that these isolates were genetically related to 1 of 4 major clades (from South America, Africa, East Asia, and South Asia) (*20*). Previous isolates from Illinois were found to be from the South America clade (*15*,*20*). The molecular epidemiology of the isolates we identified was beyond the scope of this investigation because the results of genomic sequencing were not shared with clinicians or laboratory personnel within our health system. Further research would be needed to determine whether these isolates are genetically distinct from clades previously noted to be present in the United States.

Most patients in this study were successfully treated with an echinocandin, which is consistent with treatment recommendations from the CDC (*22*). The 2 members of our cohort who died within 30 days of first positive culture attained microbiologic eradication with echinocandin therapy before they died. Three patients received azole antifungals as empiric or definitive treatment, and all 3 met our criteria for clinical success. However, treatment failures with fluconazole have been reported despite in vitro susceptibility (*8*,*21*). Echinocandin-resistant *C. auris*, possibly induced by antifungal pressure, has also been reported in the United States and in other countries (*16*,*21*). Patients with persistent or recurrent *C. auris* infections might require repeat susceptibility testing.

Several antifungal agents currently in development have activity against *C. auris*, including SCY-078, the first orally bioavailable 1,3-β-D-glucan synthesis inhibitor; VT-1598, a tetrazole-based lanosterol 14α-demethylase inhibitor; APX001, which interrupts glycosylphosphatidylinositol biosynthesis by inhibiting the fungal enzyme Gwt1; and CD101, an echinocandin that can be administered once weekly (*12*). In the future, these agents might become treatment options for *C. auris* infections, including those caused by isolates resistant to conventional therapies.

Most patients in our study who required treatment for *C. auris* were previously exposed to skilled nursing facilities and had multiple risk factors for invasive *Candida* infections, including central venous and urinary catheters (*12*). No patients were thought to have acquired *C. auris* infection during their hospital admission. The possibility of transmission within hospitals is a concern, given that *C. auris* has been shown to colonize the skin, persist on surfaces in the healthcare environment, and cause healthcare-associated outbreaks (*10*,*21*). Contact precautions, hand hygiene, and environmental cleaning and disinfection are essential to preventing the spread of *C. auris,* and these infection control practices are used within our health system (*22*).

Our study is limited by a small sample size and the inherent limitations of a retrospective, observational case series. In addition, evaluation of clinical outcomes at 30 days from the first positive culture prevented us from capturing any adverse outcomes that might have occurred after 30 days. However, our data provides insight on patient exposures. Most of these patients came from skilled nursing facilities. Targeted infection prevention and antimicrobial stewardship measures within these facilities might help to reduce the emergence and progression of resistance of this organism. Our experiences with *C. auris* are notable for favorable clinical outcomes and low rates of antifungal resistance. However, the development of resistance remains a concern, and patient response to treatment should be monitored closely.
